# Supplementary material for: Improving a data mining based diagnostic support tool for rare diseases on the example of M. Fabry: Gender differences need to be taken into account
Source: PLoS One. 2025 Jun 30;20(6):e0326372. doi: 10.1371/journal.pone.0326372 (PMC12208464; doi:10.1371/journal.pone.0326372)
Supplement: S1 File — (PDF) [file pone.0326372.s001.pdf]

Below you will find a series of questions. For each question, you can choose between **"no"**, **"rather not"** and "**no"**, **"rather yes"**, **"yes"** and **"don't know"**. Please tick **only one box** according to your answer. You can't go wrong, because there are no answers that apply to every person.

I. GENERAL:

Diagnosis (if known):

You are answering the questionnaire as the person affected ☐ You are answering the questionnaire as a relative ☐

Gender: female ☐ male ☐

Age in years: Duration of your diagnosis search in years:

Questionnaire ID (please do not fill in):

II. SEARCH FOR CAUSES:

	Possible answers:	no	rather not	rather yes	Yes	don't know
1.	Do you suspect/suspect - possibly for some time - that "something" **is wrong** with you?	☐	☐	☐	☐	☐
2.	Is it true that your complaints/irritating symptoms initially went unnoticed by doctors/your doctor?	☐	☐	☐	☐	☐
3.	If you have any abnormal test results *(e.g. blood values, ECG, skin changes, etc.)*, but the cause was **not** initially investigated further?	☐	☐	☐	☐	☐
4.	Is it true that it is/was difficult for you to put your complaints/ irritating symptoms into words?	☐	☐	☐	☐	☐
5.	Have you ever thought it possible that the cause of your complaints/ irritating symptoms lies in your lifestyle habits *(e.g. diet, living conditions, travelling, etc.)*?	☐	☐	☐	☐	☐
6.	Have you undergone a lot of tests without any results?	☐	☐	☐	☐	☐
7.	Did you or do you repeatedly go to doctors/your doctor with the same complaints/irritating symptoms?	☐	☐	☐	☐	☐
8.	Have you seen different specialists for different complaints/irritating symptoms?	☐	☐	☐	☐	☐
9.	Do you remember - in connection with your complaints - a situation that you experienced as particularly threatening?	☐	☐	☐	☐	☐
10.	Do you use special techniques *(tricks and gimmicks)* in everyday life to compensate for your limitations?	☐	☐	☐	☐	☐
11.	Do you consciously avoid activities in which your symptoms/irritating symptoms become visible?	☐	☐	☐	☐	☐
12.	Have you taken the initiative to find out about the possible causes of your complaints/irritating symptoms?	☐	☐	☐	☐	☐
13.	Have your complaints/ irritating symptoms repeatedly been given different names (diagnoses) over the course of time?	☐	☐	☐	☐	☐
14.	Did you search for doctors (specialists, experts) on your own initiative in the course of your diagnosis search?	☐	☐	☐	☐	☐
15.	Have you ever asked doctors/your doctor to carry out **examinations?**	☐	☐	☐	☐	☐
16.	Do you have the impression that the complaints you have described are/were taken seriously by doctors/your doctor?	☐	☐	☐	☐	☐
		☐	☐	☐	☐	☐

	Possible answers:	no	rather not	rather yes	Yes	don't know
**17.**	Is it true that you have lost your trust in doctors/your doctor?	☐	☐	☐	☐	☐
**18.**	Have you ever reached the point where you have given up your search for a diagnosis?	☐	☐	☐	☐	☐
**19.**	Is it true that you are/was suspected of having a mental/psychosomatic illness *(e.g. due to stress, traumatic experiences, etc.)?*	☐	☐	☐	☐	☐
**20.**	Was there a particular experience that made you particularly aware of the increase *(worsening)* of your symptoms?	☐	☐	☐	☐	☐

III. SIGNS OF ILLNESS:

	Possible answers:	no	rather not	rather yes	Yes	don't know
**21.**	Do you have/have you often had a high temperature (fever)?	☐	☐	☐	☐	☐
**22.**	Do you suffer from several complaints/ irritating symptoms at the same time *(e.g. cough **and** weight loss / skin rash **and** reduced performance **and** visual deterioration)*?	☐	☐	☐	☐	☐
**23.**	Do you suffer from constant tiredness?	☐	☐	☐	☐	☐
**24.**	Have you noticed any irritating peculiarities *(e.g. discolouration of the skin, enlargement of body parts, trembling, twitching, etc.)?*	☐	☐	☐	☐	☐
**25.**	Do you suffer/suffer from severe pain again and again?	☐	☐	☐	☐	☐
**26.**	Has your physical performance *(e.g. in sports, when climbing stairs, etc.)* noticeably decreased?	☐	☐	☐	☐	☐
**27.**	Have you noticed any breathing difficulties *(e.g. shortness of breath on exertion, severe coughing, snoring, interrupted breathing, etc.)*?	☐	☐	☐	☐	☐
**28.**	Is it true that you have an unsteady gait *(e.g. because you sway, stumble, fall, etc.)*?	☐	☐	☐	☐	☐
**29.**	Have you ever been in despair because of your symptoms?	☐	☐	☐	☐	☐
**30.**	Is it true that people around you *(family, acquaintances, friends, colleagues, etc.)* have spoken to you about physical abnormalities?	☐	☐	☐	☐	☐

IV. BRINGING COMPLAINTS UNDER CONTROL:

	Possible answers:	no	rather not	rather yes	Yes	don't know
**31.**	Have you learnt to assess your symptoms better over time?	☐	☐	☐	☐	☐
**32.**	Have you tried to alleviate your symptoms on your own initiative *(e.g. with painkillers, ointments, compresses, exercise, special diet, etc.)*?	☐	☐	☐	☐	☐
**33.**	Have you ever insisted on a certain **treatment (therapy)** with doctors/your doctor?	☐	☐	☐	☐	☐

V. BE SPECIAL:

	Possible answers:	no	rather not	rather yes	Yes	don't know
**34.**	Is it true that you can do things that others can't - or vice versa?	☐	☐	☐	☐	☐
**35.**	If your complaints/irritating symptoms only become apparent in comparison with "healthy" people clearly?	☐	☐	☐	☐	☐

	Possible answers:	no	rather not	rather yes	Yes	don't know
36.	Were you considered unathletic as a child/adolescent *(e.g. were you exempt from school sports or did not like to participate)*?	☐	☐	☐	☐	☐
37.	Are you/ were you often the centre of attention because of your complaints/ irritating symptoms?	☐	☐	☐	☐	☐
38.	Is it true that you are/was ashamed of visible changes?	☐	☐	☐	☐	☐
39.	Are your symptoms hidden or invisible to others?	☐	☐	☐	☐	☐
40.	Do you have the impression that other people have to be considerate of you?	☐	☐	☐	☐	☐
41.	Have you reduced your symptoms as much as possible in an effort to lead a normal life?	☐	☐	☐	☐	☐
42.	Is/was the uncertainty about the cause of your complaints the worst thing for you?	☐	☐	☐	☐	☐
43.	Is it true that you have ever believed yourself to be imagining your complaints?	☐	☐	☐	☐	☐

VI. SOCIAL ENVIRONMENT:

	Possible answers:	no	rather not	rather yes	Yes	don't know
44.	Do/did you have the impression that those around you *(family, friends, acquaintances, colleagues, etc.) do* **not** take your complaints seriously *(e.g. someone says: "It' s not that bad...")*?	☐	☐	☐	☐	☐
45.	Is it true that you deliberately withhold information about your complaints/irritating symptoms from those around you *(e.g. family, acquaintances, friends, colleagues)?*	☐	☐	☐	☐	☐
46.	Is it true that you prefer to stay at home *(e.g. less time with friends)? etc.)* since your complaints/ irritating symptoms became apparent?	☐	☐	☐	☐	☐
47.	Do you have a particular person you can rely on 100% in connection with your complaints/irritating symptoms?	☐	☐	☐	☐	☐

VII. EVERYDAY LIFE:

	Possible answers:	no	rather not	rather yes	Yes	don't know
48.	Do you give up certain activities that you actually enjoy?	☐	☐	☐	☐	☐
49.	Have you made any changes to your daily routine (e*.g. changed your daily routine, chosen alternative routes*) as a result of your symptoms/ irritating symptoms?	☐	☐	☐	☐	☐
50.	Is it true that you plan/planned activities *(e.g. going out, day trips, holidays, etc.)* very carefully and in advance?	☐	☐	☐	☐	☐
51.	Do you use aids to help you cope better with everyday life?	☐	☐	☐	☐	☐
52.	Are you considering a career change due to your health situation (or have you already done so)?	☐	☐	☐	☐	☐
53.	Is it true that - compared to other people - you have to make greater efforts to achieve your goals?	☐	☐	☐	☐	☐

THANK YOU VERY MUCH FOR YOUR VALUABLE SUPPORT!
