# Supplementary material for: Improving a data mining based diagnostic support tool for rare diseases on the example of M. Fabry: Gender differences need to be taken into account
Source: PLoS One. 2025 Jun 30;20(6):e0326372. doi: 10.1371/journal.pone.0326372 (PMC12208464; doi:10.1371/journal.pone.0326372)
Supplement: S2 File — (PDF) [file pone.0326372.s002.pdf]

Study number:

Study: **Improving a data mining based diagnostic support tool for rare diseases on the example of M. Fabry**

Questionnaire

Dear patient,
In addition to the "FabryScan", "Metameleon" and "Q53" questionnaires, which are used for the early detection of Fabry disease, and the "SF36" for assessing quality of life, we would ask you to answer the following supplementary questionnaire. If you have any questions, please do not hesitate to contact us!
If you need more space to answer the questions, please use the reverse side.
Thank you for your contribution!

Core data

Study number: ________________ Year of birth:___________
(This will be communicated to you by the staff of the study and will be used on all study documents instead of your name)

Gender: ☐ female ☐ male ☐ other

Highest level of education: ____________________________________

Profession: ____

Your path to the diagnosis of Fabry disease:

1. Looking back, which symptoms do you consider to be the first signs of your Fabry disease?

 ____

 ____

 ____

 ____

2. When did you experience the first symptoms of your Fabry disease?

 ____

3. On the basis of which symptoms and when did you first consult a doctor in connection with Fabry disease?

____

____

____

4. Which doctor (family doctor/paediatrician/specialist) did you consult first?

____

____

5. Were you suffering from pain in your hands or feet at the time?

☐ YES ☐ NO

6. Were your symptoms initially misdiagnosed as another illness?

☐ YES ☐ NO

If so, which one?

____

____

7. How many doctors did you consult in connection with Fabry disease before your diagnosis?

____

8. Have you ever requested that certain diagnostic tests be carried out?

☐ YES ☐ NO

If yes: Which kind?

____

____

____

____

9. Did you try to gather information about your illness on the Internet before you were diagnosed?

☐ YES ☐ NO

If so, what keywords did you enter into search engines?

____

____

____

____

____

10. When was Fabry disease diagnosed?

____

11. Are you undergoing treatment for Fabry disease?

☐ YES ☐ NO

If yes: Which one and since when?

____

____

____

12. Which doctor (family doctor/paediatrician/specialist) initiated the investigations that led to your diagnosis?

____

____

13. Do you have relatives or acquaintances who are not related to you with Fabry disease?

☐ YES ☐ NO

If yes: Which ones (parents/siblings/children/friends/neighbours etc.) and when were they diagnosed?

____

____

____

____

Your opinion on diagnostic tools such as Q53 and FabryScan

14. Do you welcome the use of tools such as Q53 and FabryScan to to help diagnose rare diseases?

☐ YES ☐ RATHER YES ☐ RATHER NO ☐ NO

15. What concerns or hopes do you associate with the use of computer-aided diagnostic procedures such as Q53?

____

____

____

____

16. Does the use of diagnosis-supporting procedures such as Q53 and FabryScan reflect a lack of expertise on the part of the doctor who uses them?

☐ YES ☐ RATHER YES ☐ RATHER NO ☐ NO

17. Who should use diagnostic support procedures such as Q53 and FabryScan?

____

____

____

18. How long did it take to complete the questionnaires? Please estimate retrospectively:

Q53 ca. ____________ Minuten
FabryScan ca. ____________ Minuten
Metameleon ca. ____________ Minuten

19. Were all the questions in questionnaire Q53 formulated clearly and comprehensibly?

☐ YES ☐ NO

If no: Which questions were not phrased clearly enough?

____

____

____
