# Supplementary material for: Improving a data mining based diagnostic support tool for rare diseases on the example of M. Fabry: Gender differences need to be taken into account
Source: PLoS One. 2025 Jun 30;20(6):e0326372. doi: 10.1371/journal.pone.0326372 (PMC12208464; doi:10.1371/journal.pone.0326372)
Supplement: Attachment — Submitted filename: Response to Reviewers_11.05.25.docx [file pone.0326372.s005.docx]

Bochum, 03.05.2025

Dear Editor, Dear Reviewers,

We are pleased to receive your response and sincerely appreciate your consideration of our manuscript for publication in *PLOS ONE*.

We are deeply grateful for your insightful comments, your careful attention to detail, and the time you have devoted to reviewing this complex subject. Your suggestions have significantly contributed to improving our work, and they will also inform and enrich our future research.

We have carefully addressed each of the points you raised and have revised the manuscript accordingly to correct the identified weaknesses. Please find our detailed responses to your comments below.

Major concerns:

Reviewer comment:
The authors have described a need for diagnostic assistance in Fabry disease, as early diagnosis is important for the successfully use of therapies in these patients. The authors acknowledge that all participants in the study knew their diagnosis, in some cases for years, and they may not have answered the questionnaire in the same way as they would have before their diagnosis. However, they do not discuss how this aspect of the training data may affect the utility of their model in diagnosing new patients with Fabry disease.

Reply: A notable limitation of our study is that the diagnostic support tool (the questionnaire) was developed using data from individuals who were already aware of their Fabry disease diagnosis, in some cases for many years. This awareness may have influenced how participants recalled and reported their symptoms, potentially introducing recall bias or confirmation bias. Specifically, individuals might emphasize symptoms that they now recognize as characteristic of Fabry disease or minimize symptoms they retrospectively deem unrelated. This could result in a pattern of responses that differs from how individuals with undiagnosed Fabry disease would naturally answer the same questions.

Consequently, the questionnaire's predictive performance may be artificially optimized for individuals who have already internalized their diagnostic label, rather than reflecting the symptomatology of individuals in the pre-diagnostic phase. We recognize that this may limit the generalizability of the tool in real-world diagnostic settings, where patients are unaware of their diagnosis and may present with less structured or less disease-specific symptom reports. For this reason we added the following paragraph on page 24 lines 8-19: *“Additionally it has to be taken into account that all participants knew their diagnosis and some of them had been dealing with the disease for years. In particular, the recruitment of participants via the patient organization may have contributed to reaching particularly committed and well-informed participants. Their knowledge of the disease may have influenced how participants recalled and reported their symptoms, potentially introducing recall bias or confirmation bias. Specifically, individuals might have emphasized symptoms that they now recognize as characteristic of Fabry disease or minimized symptoms they retrospectively deem unrelated. This could result in a pattern of responses that differs from how individuals with undiagnosed Fabry disease would answer the same questions. Consequently, the CAD system’s predictive performance may be optimized for individuals who have already internalized their diagnostic label, rather than reflecting the symptomatology of individuals in the pre-diagnostic phase.”*

Future validation efforts should therefore include prospective studies involving individuals suspected of Fabry disease but not yet diagnosed, to assess the tool’s performance in a diagnostically naive population as commented in the manuscript on page 24, line 21 – page 25, line 2: *“To address this, we recommend in future work a prospective validation in undiagnosed populations, refinement of the questionnaire to prioritize symptom-based rather than journey-based items, and gender-specific calibration to ensure equitable diagnostic support. This approach will help ensure that the tool fulfills its goal of facilitating earlier and more accurate diagnosis across the full clinical spectrum of Fabry disease.”*

Such validation would help determine the true clinical utility of the CAD system for early detection and reduce the risk of overestimating its diagnostic accuracy, which was critically acclaimed in the manuscript on page 26, line 9-12 by adding: “*Future validation efforts should include prospective studies involving individuals suspected of Fabry disease but not yet diagnosed, to assess the tool’s performance in a diagnostically naive population. Such validation would help determine the true clinical utility of the CAD system for early detection and reduce the risk of overestimating its diagnostic accuracy.”*

Reviewer comment:
Additionally, given that many of the sex-specific differences in answers to the questionnaire, I am particularly concerned that the tool described here cannot effectively be used to aid in the diagnosis of new patients, particularly for women. Of the questions with diametrically opposed answers between men and women, many of the women’s answers indicate a long and difficult journey before their diagnosis. If this is the data used to train the model, can the model aid in the diagnosis of new patients if they have not also experienced things like getting different names (diagnosis) for symptoms over time (question 13), giving up on a search for a diagnosis (question 18), a psychological or psychosomatic disease being suspected (question 19). If this tool is meant to aid in earlier diagnosis of Fabry disease, I believe the authors should eliminate survey questions whose answers refer to a long or challenging diagnostic odyssey from their model.

Reply: An important concern raised by the reviewer relates to the sex-specific differences observed in responses and the potential implications for the questionnaire's ability to aid in early diagnosis, particularly in women. We acknowledge that several questionnaire items, including those related to experiences of a prolonged diagnostic journey (e.g., receiving multiple different diagnoses over time, abandoning the search for answers, or being suspected of psychosomatic illness), reflect consequences of delayed diagnosis rather than early disease manifestations. Women with Fabry disease, who are often diagnosed later and may present with more heterogeneous or less classical symptoms compared to men, were more likely to endorse these items.

As such, including these "diagnostic odyssey" questions in the development of the tool risks reinforcing a late-diagnosis profile, potentially limiting the questionnaire’s sensitivity for identifying patients earlier in their disease course — precisely the population in whom early therapeutic intervention would be most beneficial. We recognize that an effective diagnostic support tool should ideally focus on symptoms and clinical signs present early in disease progression rather than the sociomedical consequences of delayed recognition and added the following paragraph in the discussion section (page 23 line 19 – page 24 line 7): *“Still, while our model offers a promising approach to capturing patient-reported characteristics of Fabry disease, the inclusion of these "diagnostic odyssey" questionnaire items, which capture the real-world burden of delayed diagnosis, in the development of the tool risks reinforcing a late-diagnosis profile and potentially limiting the sensitivity of the system to identify patients earlier in their disease course, particularly women. An effective diagnostic support tool should ideally focus on symptoms and clinical signs present early in disease progression rather than the sociomedical consequences of delayed recognition. All questions origin from interviews reflecting the time and symptoms prior to diagnosis. No question should therefore be regarded as “survey question”. Nevertheless, diagnostic delay, symptom burden, psychosocial stressors, and individual resilience all influence the pattern of response—beyond the underlying disease itself. After all, the questions used here were developed from interviews with individuals affected by a wide range of rare diseases, who consistently reported similar experiences during the pre-diagnostic phase of their condition.”*

One aspect that gives hope that the tool may be useful at this stage for patients who have not undergone a long diagnostic odyssey is the following observation, which we were pleased to note during the analysis but have only now included in the article (page 22) at the suggestion of the reviewer: While the mean delay from first symptoms to diagnosis (15.6 years for female participants, 20.5 years for male participants) and the mean diagnostic journey (time from first consultation to diagnosis, 7.5 years for females, 6.5 years for males) for all participants were longer than those reported by Reisin et al. in 2017 (Time delays in the diagnosis and treatment of Fabry disease, Reisin et al. 2017), four of the female participants were diagnosed based on incidental findings by an ophthalmologist or family examination before they had any recognisable symptoms. These participants were also reliably identified by the CAD system and, with average scores of ~0.92 for 'Fabry' and ~0.05 for 'Other', even outperformed the overall participant average (~0.90 for 'Fabry' and 0.07 for 'Other').

In response to this valid concern, we propose that future iterations of the tool will re-evaluate the weighting or inclusion of items that primarily capture the experience of diagnostic delay rather than disease-specific symptomatology and added this concern on page 26 lines 5-8. In particular, analyses stratified by sex will be essential to ensure that the tool performs equally well in men and women. Furthermore, a prospective validation cohort including individuals prior to formal diagnosis could provide critical data to refine the tool, enabling the identification of questions most predictive of Fabry disease without reliance on retrospective diagnostic experiences.

Ultimately, while the current questionnaire provides valuable insight into the patient journey, careful consideration and possible recalibration will be required to optimize its utility for early diagnostic support across sexes and clinical presentations.

Reviewer comment:
If this is not feasible, the authors should at least add a more robust discussion of the potential impacts of the structure of the participant data on the ability of their model to aid in diagnosis. Several of the authors have expertise in the clinical diagnosis of rare diseases and their perspective on this aspect of the model will provide important context to their work.
Since several of the authors have extensive expertise in clinical diagnosis of rare diseases, I ask the authors to add a more robust discussion of the potential impacts of the structure of the participant data on the usefulness of their model in aiding diagnoses, both in general and for women and men individually, given not only the fact that participants already know they have the disease, but that many seem to have a long diagnostic odyssey and are likely to answer a number of the survey questions very differently from those early in their search for a diagnosis.

Reply: We appreciate the reviewer’s request for a more detailed discussion regarding the influence of the participant data structure on the potential diagnostic utility of our model. As acknowledged, all participants in our study were already diagnosed with Fabry disease at the time of completing the questionnaire, and many had experienced a protracted and complex diagnostic journey. These factors introduce several important considerations regarding the generalizability and application of the tool, both in general and across sexes.

First, the retrospective nature of data collection, where participants reflect on their experiences with full knowledge of their diagnosis, may lead to differential symptom reporting compared to individuals who are undiagnosed and early in their diagnostic trajectory. Participants may be more attuned to disease-specific symptoms in hindsight, and their responses may also reflect cognitive biases related to diagnostic confirmation or medical experiences following their diagnosis. As a result, the model may preferentially capture characteristics of individuals who have already undergone extensive medical evaluation rather than those presenting earlier with less defined symptomatology. We highlighted this limitation in our discussion by adding the sentences on page 24, lines 11-19.

Second, given the sex-specific diagnostic patterns observed in Fabry disease — where men often present earlier with more classical features, while women may exhibit subtler or more heterogeneous manifestations — there is a heightened risk that the model's predictive capability may be uneven between sexes. In particular, several questionnaire items reflect experiences of misdiagnosis, psychological labeling, or diagnostic abandonment, which were endorsed more frequently by women. We acknowledged this on page 23, lines 5-10. These items, while capturing the real-world burden of delayed diagnosis, may be less useful for identifying patients at an earlier stage of disease.

Therefore, while our model offers a promising approach to capturing patient-reported features of Fabry disease, its current structure may be more reflective of a "post-diagnosis" or "late-diagnosis" phenotype, particularly in women A discussion of this limitation is now included in the discussion section (page 23, lines 19-23): *“Still, while our model offers a promising approach to capturing patient-reported characteristics of Fabry disease, the inclusion of these "diagnostic odyssey" questionnaire items, which capture the real-world burden of delayed diagnosis, in the development of the tool risks reinforcing a late-diagnosis profile and potentially limiting the sensitivity of the system to identify patients earlier in their disease course, particularly women.”* To address this, we recommend in future work a prospective validation in undiagnosed populations, refinement of the questionnaire to prioritize symptom-based rather than journey-based items, and gender-specific calibration to ensure equitable diagnostic support. This approach will help ensure that the tool fulfills its goal of facilitating earlier and more accurate diagnosis across the full clinical spectrum of Fabry disease, as stated in the revised version of the manuscript on page 24, line 21 – page 25, line 2.

Reviewer comment:
The authors emphasize the importance of sex-specific differences in both the expression of symptoms and disease perception. It is logical to distinguish male and female participants in a study of an X-linked disorder. But the authors show no data to indicate that distinguishing between male and female participants improves the performance of their model. Indeed, on page 21 they state, “we can only surmise the influence of the distinction between men and women on the improvement in performance,” but again show no data about this improvement in performance. If they wish to make these assertions, the authors should compare the AI as currently trained to account for sex-specific differences to a model that does not separate male and female participants. How much does the separation of sex-specific differences improve the algorithm’s specificity in detecting Fabry disease?

Reply: Our study emphasizes the biological and clinical relevance of considering sex-specific differences in Fabry disease, an X-linked disorder where symptom presentation, severity, and diagnostic delay differ substantially between men and women. Recognizing these differences is essential for clinical interpretation; however, we acknowledge the reviewer’s important point that our manuscript does not provide direct comparative data demonstrating that separating male and female participants improves the performance of the diagnostic support tool.

Indeed, while we hypothesized that accounting for sex-specific differences could enhance model specificity and sensitivity — particularly given the more heterogeneous and frequently milder phenotypes observed in women — we did not perform a formal comparison between models trained with and without sex distinction. Therefore, our current assertion remains speculative rather than evidence-based, as stated on page 22, lines 1-7: *“At the same time, as we do not have any comparable pre-training data on the performance of the system on FD, especially not regarding its performance without the distinction of female and male patients, we can only assume rather than prove the influence of this distinction between men and women on the improvement in performance. However, fundamental differences in disease processing and the clearly distinct response patterns observed in this study suggest that incorporating gender-specific disease models may also enhance the sensitivity of CAD systems for other diseases.”*

.

To fully substantiate the role of sex-specific stratification in improving diagnostic accuracy, future work should involve explicit testing of two modeling approaches: (1) models trained with pooled data regardless of sex, and (2) models incorporating sex as a separate feature or developing sex-specific models. Direct comparison of the performance metrics (e.g., sensitivity, specificity, positive predictive value) between these approaches will be crucial to determine whether stratification materially enhances early detection across both sexes, particularly for women who historically experience greater diagnostic challenges. To underline this aspect and the valuable hint of the reviewer we added two sentences on page 22, lines 8-14.

Until such comparative analyses are performed, we recognize that our claims regarding the benefits of sex-specific modeling must be framed cautiously. In future studies, we aim to rigorously evaluate and report the impact of sex-specific modeling choices on algorithmic performance in prospective and external validation cohorts.

Reviewer comment:
Furthermore, given the disparity between the number of Included female and male participants with Fabry disease in the training set, do the authors see significant differences in the sensitivity of the AI for detecting Fabry disease in women compared to men. Any significant differences would be important information if this model is to be used clinically as well as for informing future directions for improving the model.

Reply: It is a fair assumption that the CAD is more sensitive to the presence of FD in women than in men because significantly more data sets were available for women. The CAD correctly assigned all participants in this study, so a direct conclusion is not possible, but an approximation to this question could be made by looking at the average difference between Fabry score and other score for men and women: Scores range from 0 to 1. As long as the Fabry score is higher than the other score, the CAD correctly recognizes the FD patients. On average, the Fabry scores of women are 0.86 higher than the other scores, while for men the difference is 0.75. This difference can in fact be attributed to the different number of training data. It would undoubtedly be desirable to train the CAD with further data in the future, which would most likely increase the difference in the scores. However, it is not possible to reliably deduce from this how much higher the probability is for a male Fabry patient to erroneously be assigned a higher other score than his Fabry score. We cannot deduce a clinically significant difference in the application of the CAD from our observations in this study.
A paragraph addressing this issue has been added (page 22, line 16-20).

Reviewer comment:
The description of the stratified cross-validation is unclear and confusing. Particularly this sentence on page 11, “In each of the eleven runs, other randomly selected eight of the 88 data sets were not used to train the AI but were used for subsequent testing.” It would be helpful to more clearly and explicitly describe the overall data set used for cross validation and the approach to the stratified selection.

Reply: We gladly replaced this indeed confusing sentence with a more detailed explanation and inserted a sentence a little further up the page that we hope concisely summarizes the preceding description of the test data set (page 11).

Reviewer comment:
On page 20, the authors state that their algorithm can indicate a rare disease without a pre-formulated suspected diagnosis, however, it was trained and tested on individuals who already had a Fabry disease diagnosis. It is unclear to me that the authors can make this claim. If they can they need to more clearly explain why this claim is valid.

Reply: The sentence was indeed unfortunately worded in a misleading way. It referred to the remarks on the comparison with FabryScan on page 19. What we have tried to write is that it is not necessary to consider the differential diagnosis of FD prior to the application of the CAD in order to obtain an indication of the presence of FD through the tool. In this respect, it differs in particular from a tool like FabryScan or from targeted Fabry diagnostics.
We have therefore sought to replace the term "pre-formulated" with a clearer term (page 20).

Minor concerns:

Reviewer comment:
The authors use the terms “gender” and “gender differences” but I believe the differences they are referring to are a result of differences between biological sexes in experiencing an X-linked disorder, and thus the terms “sex” and “sex-specific differences” should be used instead. Please make this change to reflect the more accurate terminology.

Reply: We thank the reviewer for this thoughtful observation. We fully acknowledge that Fabry disease is an X-linked condition and that the underlying biological basis of the differences observed is rooted in genetic sex. We have therefore changed the term on page 21, line 12, where it referred to the difference in symptom expression between male and female patients.
However, we have chosen to primarily use the term “gender” in our manuscript in a broader sense, reflecting not only biological differences but also how individuals experience disease within healthcare systems and society, where the concept of gender can influence diagnosis, treatment, and outcomes.

While “sex” would be more strictly accurate from a biological perspective, our usage was intended to encompass both biological and social dimensions. We have therefore retained the original terminology but have added a brief clarification in the manuscript to acknowledge this distinction (page 4, lines 10-13). We hope this addresses the reviewer’s concern while preserving the intended scope of our discussion.

Reviewer comment:
Please provide a more detailed explanation of the “physical sum scale.” From the context provided in the text I believe it quantifies the answers to the quality of life survey, but this should be explicitly described.

Reply: It is entirely true that these explanations were accidentally completely neglected. The reviewer's assumption is indeed correct. To ensure that this is comprehensible to other readers as well, we have added an explanation in the Methods section (page 7, lines 19-22).

Reviewer comment:
In figure 1, please include a label or legend in the figure to make it easy to know which color denotes answers from male participants and which are from female participants to improve readability of the figure.

Reply: We incorporated two additional explanatory labels (page 13, line 18) into the image to enhance its clarity and comprehensibility.

Reviewer comment:
Please provide more detailed figure legends. For example, in Figure 1, there are some horizontal black lines which have unclear significance. If these lines do not have significance, please remove them from the figure. For Figure 2, please provide more plain English detail of the comparisons or calculations done to generate the ROC curve.

Reply: We gladly revised the figure legends and have amended the caption of the Figure 1 in particular to include an explanation. We felt it would be helpful to add the previously missing more detailed explanation of the data basis and process of generating the ROC curve in the text rather than in the caption of Figure 2. Therefore, the explanations on page 12 have been reworded and expanded accordingly. There has also been a corresponding extension to the explanation on pages 15-16.

Reviewer comment:
There is a typo on page 16: “KI-based approaches for rare diseases” should be “AI-based approaches for rare diseases.”

Reply: the manuscript was revised accordingly.

Reviewer comment:
The discussion of the commonly used algorithms in CAD systems for rare diseases does not add to the discussion section of this manuscript. This paragraph seems to address the reasoning behind why the classifier systems used in this algorithm were selected and seems better suited to an introduction section.

Reply: Since this paragraph not only provides a placement of the CAD in the context of currently used methods, but also gives an otherwise somewhat neglected insight into how it works, we were very happy to move it to the section ”Development of the AI-based computer-diagnosis system" (page 5, line 18-25)

We gladly correct the **Competing Interests statement** by adding:
I have read the journal's policy and the authors of this manuscript have the following competing interests: LG and WL are co-founders of the commercial company, Improved Medical Diagnostics IMD GmbH. The authors are not employees of Improved Medical Diagnostics IMD GmbH, nor are there other relevant declarations relating to consultancy, patents, products in development or marketed products, etc. The co-foundation of these authors (LG, WL) does not alter our adherence to PLOS ONE policies on sharing data and materials. All other authors have declared that no competing interests exist. This does not alter our adherence to  PLOS ONE policies on sharing data and materials.

Additionally, the reference to the complete **ethics statement** has now been added, as well as the indication of the written consent obtained from each participant (page 8, lines 10-15).

We would like to once again thank the reviewers for their thoughtful, positive and constructive comments, which have greatly contributed to improving our manuscript. We hope that the revised version meets your expectations and look forward to a positive decision regarding its publication.

With kind regards,

Philipp Hahn
